# Microfluidic Gut-on-a-Chip: Fundamentals and Challenges

**DOI:** 10.3390/bios13010136

**Published:** 2023-01-13

**Authors:** Dimple Palanilkunnathil Thomas, Jun Zhang, Nam-Trung Nguyen, Hang Thu Ta

**Affiliations:** 1 Queensland Micro- and Nanotechnology, Griffith University, Nathan, QLD 4111, Australia; 2 School of Environment and Science, Griffith University, Nathan, QLD 4111, Australia; 3 Australian Institute for Bioengineering and Nanotechnology, The University of Queensland, St. Lucia, QLD 4072, Australia

**Keywords:** lab-on-a-chip, organ-on-a-chip, gut-on-a-chip, microfluidics, human gut

## Abstract

The human gut is responsible for food digestion and absorption. Recently, growing evidence has shown its vital role in the proper functioning of other organs. Advances in microfluidic technologies have made a significant impact on the biomedical field. Specifically, organ-on-a-chip technology (OoC), which has become a popular substitute for animal models, is capable of imitating complex systems *in vitro* and has been used to study pathology and pharmacology. Over the past decade, reviews published focused more on the applications and prospects of gut-on-a-chip (GOC) technology, but the challenges and solutions to these limitations were often overlooked. In this review, we cover the physiology of the human gut and review the engineering approaches of GOC. Fundamentals of GOC models including materials and fabrication, cell types, stimuli and gut microbiota are thoroughly reviewed. We discuss the present GOC model applications, challenges, possible solutions and prospects for the GOC models and technology.

## 1. Introduction

Advances in microfabrication and micromachining have enabled lab-on-a-chip technology that can manipulate fluid flow at the microscale and integrate many biochemical analyses on a single miniaturised device. Lab-on-a-chip technology has been used for research areas such as molecular biology (e.g., DNA analysis), cellular biology (e.g., stem cell research and diagnostics), proteomics (e.g., protein analysis) etc. Laboratories over the past years have focused on the advancement and utilisation of these lab-on-a-chip devices due to their compactness, low cost, ease of use, high parallelisation, real-time processing and monitoring, increased sensitivity etc. The combination of lab-on-a-chip and bioengineering leads to organ-on-a-chip (OoC) technology, a sophisticated microfluidic cell culture system designed to replicate complex tissues with their structures, functions, physiology, and pathologies. Such systems can mimic organ-level pathophysiology *in vitro*. With the help of OoC, researchers have been able to study tissue–tissue interfaces and intricate organ-specific chemical and mechanical microenvironments, creating *in vivo*-like complex tissues such as the liver [1,2], brain [3], heart [4,5,6], kidney [7,8] and intestine [9,10,11].

**Figure 1:**
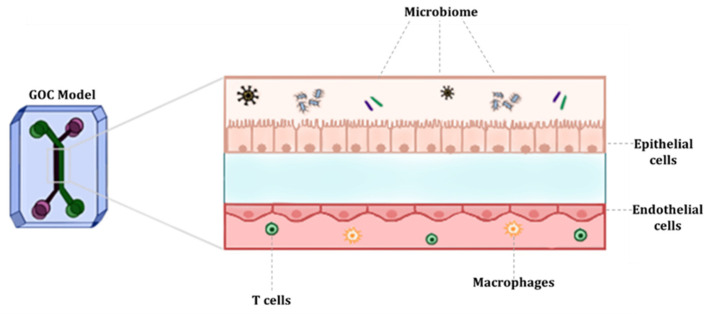
Schematic representation of GOC model mimicking the microenvironment of the gut.

The human gut has gained significant interest recently due to its vital role in the digestion and absorption of nutrients. The gut is also known to play a crucial part in sustaining the function of other organs and in the aetiology and pathogenesis of various diseases. Although the human gut plays an integral role in maintaining homeostasis, a major knowledge gap still exists on its mechanisms and host-microbe interactions because of the lack of appropriate models. Previously commonly used models, animal models, cannot accurately represent human physiology and its responses to pathogens, diseases and drugs. To overcome these barriers, researchers over the past decade have developed and implemented *in vitro* and *ex vivo* models that are capable of appropriately representing the human gut, known as gut-on-a-chip (GOC) models, which are used to study the gut physiology in a lab-on-a-chip format (Figure 1). However, some of these models fail to appropriately express the physiological processes and parameters the human gut undergoes due to the intricacy of its structures, components, and functions [12].

The majority of these *in vitro* models use two-dimensional (2D) cell culture, which is also limited as it does not appropriately represent the three-dimensional (3D) structure of the organ or surrounding tissues. Thus, 3D models are more promising for appropriately mimicking the physiology and pathology of GOC. Innovations in microfluidics and microfabrication have accelerated the advancement of GOC models and have been extensively used for biological research [13,14]. Over the past decade, reviews published focused more on the applications and future prospects of GOC technology, but the challenges and solutions to these limitations were often overlooked. In this review, we provide a thorough overview of the physiology and distinctive characteristics of the human gut. We also discuss the latest development of GOC models including the fundamentals of these GOC models, applications, challenges with respective solutions and potential applications in the biomedical science field.

## 2. Characteristics and Physiology of the Human Gut

**Figure 2:**
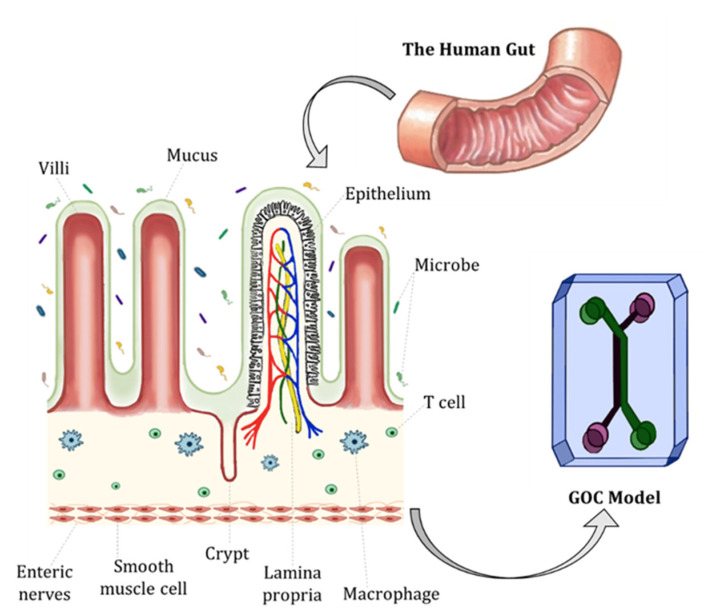
The schematic representation of the human gut in a GOC model. The human gut is covered with finger-like villi structures and invaginations called ‘crypts of Lieberkühn’.

The human gut, also known as the digestive or gastrointestinal (GI) tract, is a series of connected organs, which extends from the mouth to the anus. The main role of the human gut is food digestion, nutrient absorption, fluid homeostasis and secretion of waste. The small intestine is where the majority of food digestion and nutrient absorption mechanism occurs. The small intestine is lined with permanent folds known as plicae circulares. Each plica is covered by numerous tiny finger-like projections called villi, which are circular folds of mucosa and submucosa. Each villus has several microvilli, further increasing their surface area. The villi are a distinctive feature of the small intestine; villi are responsible for absorbing digested food and transporting nutrients into capillaries as seen in Figure 2 [15,16].

The large intestine, on the other hand, is responsible for osmosing water and electrolytes. Moreover, undigested food which progresses into the large intestine is broken down by microbiota residing in the large intestine. The human gut harbours a large variety of microbiomes, estimated at ~100 trillion, between 500 and 1000 species, consisting of bacteria, yeasts, and parasites [17]. The gut microbiota is vital for maintaining homeostasis and nutrient absorption, regulating the intestinal epithelial mucosal barrier, and protecting against pathogens and drug metabolism. Changes in the composition of the gut microbiome, due to diet or medication, disrupts homeostasis and can induce pathogenesis [18]. Moreover, there are five prominent families of intestinal flora which are Actinobacteria, Bacteroidetes, Firmicutes, Proteobacteria and Verrucomicrobia [19,20]. Some of previously noted ‘beneficial’ bacteria are Lactobacillus Acidophilus and various species of Bifidobacterium often seen in probiotic supplements.

Other structures and features essential for maintaining homeostasis in the intestine are the epithelium and mucosa. The intestinal epithelium comprises a single layer of diverse intestinal epithelial cell (IEC) types including absorptive enterocytes (i.e., the most pronounced and common cell type in the intestinal epithelium), neuroendocrine cells, tuft cells, goblet cells, Paneth cells and microfold (M) cells. The base of crypts is where intestinal stem cells reside, which give rise to proliferative stem cells that differentiate and migrate upwards to form the epithelial layer. The integrity of the intestinal epithelium is vital for maintaining gut homeostasis and acts as a barrier against pathogens [16].

Furthermore, different types of barriers are generated by IECs to shield the intestinal mucosa from the invasion of pathogenic organisms or commensal microbes. These barriers are divided into two main subtypes, physical and chemical barriers. Physical barrier examples include the intestinal mucosa that prevents the invasion of pathogens. The mucosal layer creates a frontier limiting the contact between the intestine and pathogens. The thickness of the mucosal layer varies between regions of the GI tract [21]. For instance, the small intestine has a very thin single lining of mucus (~20 µm) which is facedly adherent to the epithelium and is also easily permeable. Whereas, in regions such as the distal colon the thickness of the mucus layer is much greater (~830 µm), and there are two mucus layers (a stratified adherent inner mucus lining and outer mucus layer that is less defined) resulting in a firmer adhesion to the epithelium. Therefore, the small intestine has a greater presence of chemical barriers such as antibacterial and immune modulators compared to the colon which has a thicker mucus layer [22,23]. Chemical barriers include antimicrobial peptides (AMPs), which are amino acid-rich proteins that are part of the innate immune response. AMPs attach to the microbial cell membrane and disrupt the membrane of invading pathogens by forming pore-like structures. These barriers assist in maintaining the symbiotic relations of commensal microbes, IECs and immune cells [24].

**Figure 3:**
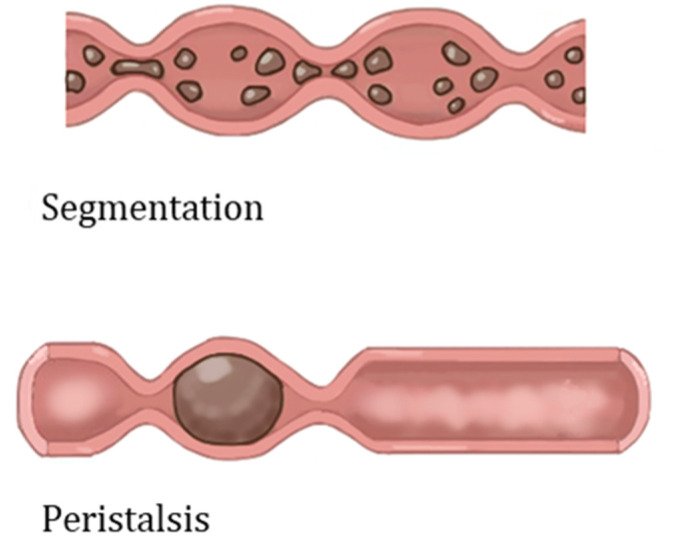
The schematic of peristaltic and segmentation contractions of the GI tract [27].

Moreover, the epithelium is surrounded by layers of smooth muscles with enteric neural systems rooted within muscles that regulate intestinal mechanical stimuli. The stimuli the human intestine experiences are peristaltic and segmental contractions (Figure 3), which is responsible for the movement of food through the GI tract [16]. Both stimuli assist in the movement and absorption of foods. Peristaltic movement is a one-way involuntary movement that facilitates food propulsion, achieved by a series of relaxation and contraction of the circular smooth muscle. Specifically, adjacent sections of the alimentary canal organs consecutively contract and relax, minimally mixing food but moving distally through the GI tract. Peristalsis results in the high-speed propagation of food. Segmentation, on the other hand, is a forward and backward motion, where rhythmic contractions of longitudinal muscles occur. Compared to peristaltic motion, segmentation allows for the thorough mixing of food and is a slow propagation of food [25,26].

Another unique characteristic of the human gut is the steep oxygen gradient, whereby different regions of the GI tract reflect different partial pressures (pO_2_). Interestingly, under normal conditions, it is understood that the intestinal epithelium exists in a state known as ‘physiological hypoxia’ [28]. At sea, the pO_2_ of air is ~145 mm Hg (~21% O_2_), through non-invasive methods such as electron paramagnetic resonance (EPR) oximetry, it is estimated that in tissues across the colonic muscle wall the pO_2_ is ~42–71 mm Hg (~7–10% O_2_) to around ~11 mm Hg (~2% O_2_) in the lumen of the ascending colon, ~42 mm Hg (~6% O_2_) in vascularised submucosa, 59 mm Hg (8% O_2_) in the small intestinal wall and ~22 mm Hg (3% O_2_) at the villi tip [29]. These environments are created due to the high-energy requirements of the gut for dietary nutrient breakdown and metabolism, maintenance of homeostasis and oxygen intake of microbiota allowing them to proliferate and maintain the lumen in a deeply anaerobic state. This state of hypoxia induces the production of hypoxia-induced factors (HIFs) by epithelial cells. Upon stabilisation, HIFs regulate gene expression which is crucial for energy metabolism, barrier integrity and angiogenesis [29,30].

## 3. Fundamentals of GOC Models

Recent progress in microfluidic technology has made it possible to mimic characteristics and responses of the human gut as seen *in vivo*. In the past decade, researchers have enhanced GOC models by incorporating sensors and biometers to control parameters that mimic the human gut. Most *in vitro* GOC models depend upon 2D cell culture models, whereby the intestinal epithelial cell lines (i.e., human colon adenocarcinoma (Caco-2) or human colorectal adenocarcinoma cell line with epithelial morphology (HT-29 cells)) are grown on ECM-coated porous membranes inside Transwell systems or 2D monoculture plates. These models are often used to study the barrier functions and drug absorption; hence they are primarily applied in the pharmaceutical industry. 2D Transwell culture systems are simple and can be used for short-term observations; however, they fail to recapitulate the 3D structures and interactions of the native tissue such as microstructures (i.e., microvilli), mucus production, peristaltic motion, drug metabolism, etc. Another challenge with conventional models is due to the static nature the integration of commensal microbiomes, such as bacteria (i.e., *E. coli*) due to overgrowth and contamination of the system [31].

To appropriately study the human gut’s physiology, pharmacology or pathology, the system used must recreate the 3D structures and microenvironment of the human gut. This can be achieved by using microfluidic platforms and incorporating live cells, thereby creating a 3D model with dynamic cell culture, and overcoming challenges such as microbial overgrowth [14,32]. The most common GOC model structure has two channels (upper and lower layer), separated by a porous semipermeable membrane, which depicts the separation between the intestinal lumen and the vasculature. Furthermore, one of the two microchannels represents the lumen of the human gut. This channel aligns with the gut epithelial cells (i.e., IECs). The other channel represents the blood vessels and therefore aligns with vascular endothelial cells. The role of the semipermeable membrane is to facilitate the transport of soluble molecules and nutrients between the gut and the blood vessels [33].

Moreover, researchers have highlighted vital characteristics which are essential to represent the human gut appropriately and successfully in GOC models including: (i) peristalsis-like motions, (ii) mimicking the structure of villi and (iii) creating an oxygen gradient. Other relevant features vital to reproducing the human gut on GOC models include creating an intestinal barrier and applying shear stress and mass transport. The inclusion of these features in GOC models allows for accurately mimicking the physiological factors experienced by the human gut. Measuring and monitoring these parameters are also crucial to ensure that the physiological factors are met in models. The parameters usually monitored and measured are barrier permeability, dissolved oxygen levels and cytokine production. Table 1 highlights previous GOC models and their respective technological approaches and characteristics [15].

### 3.1. Materials and Fabrication

**Figure 4:**
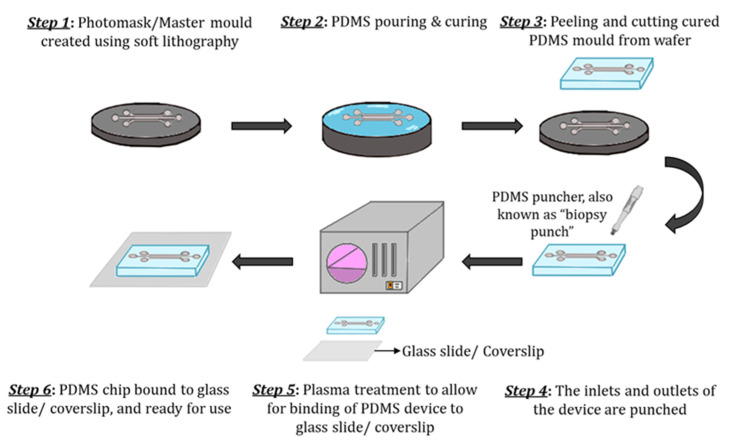
Schematic of the step-by-step process of PDMS device fabrication. (**Step 1**) The master mould is created using soft lithography. (**Step 2**) The PDMS mixture (10:1 (base: curing agent)) is poured over the master mould and cured. (**Step 3**) The solidified PDMS is then cut and peeled from the master mould. (**Step 4**) The inlets and outlets are punched using a biopsy puncher. (**Step 5**) The PDMS chip and glass slide/coverslip are activated through plasma treatment. (**Step 6**) Finally, the PDMS chip is bound to a glass slide/coverslip and cured further for ~2–5 min at 75 °C and is ready to use.

The most common material used for microfluidic device fabrication is polydimethylsiloxane (PDMS). PDMS has unique characteristics such as low elasticity, chemical inertness, high electrical resistance, porosity, and non-toxicity [39,40]. PDMS has been widely used because of its low costs and accessibility, optical transparency and gas permeability. PDMS has a refraction index of 1.4, allowing it to be compatible with various optical imaging and analysis methods. One can make pores with sizes ranging from 2 to 10 µm in PDMS. However, one of the major disadvantages of using PDMS for GOC models is the hydrophobicity, consequently leading to the absorption of lipophilic compounds [41]. Inkjet printing and soft lithography have been used to make PDMS microfluidics devices. Soft lithography is the most used method having two essential steps, photolithography and replica moulding. Briefly, the fabrication process of PDMS-based GOC models is as follows (Figure 4). Liquid PDMS prepolymer is mixed with a curing agent, in a standard weight ratio of 10:1 (base: curing agent). The combined PDMS is poured over a master mould and then placed in a desiccator. The PDMS is then cured for one hour at 75 °C. After curing, PDMS can be carefully peeled from the master mould, and the inlet and outlet holes are punched [42]. The PDMS chip is then washed and dried, ready for plasma treatment. After plasma binding, the PDMS chips are bound to the glass slide/coverslip and incubated for ~2–5 min at 75 °C. The semipermeable membrane, which separates the microchannels, is usually composed of PDMS, polycarbonate (PC), polyester (PE), polyethylene terephthalate (PET) and polytetrafluoroethylene (PTFE). Moreover, biological materials, such as collagen can also be used. To increase the biocompatibility of some materials, membranes and microchannels are coated with natural polymers such as extracellular matrix (ECM) proteins such as collagen, fibronectin and gelatin, as they provide natural moiety for cell adhesion and survival [12,42,43,44]. Although PDMS is the commonly used material, it is unfavourable to cell adhesion, and to address this issue, channels are coated with hydrogel.

Hydrogels are a polymeric material that has been used to fabricate OoC and GOC models. Hydrogels have a high-water content and can mimic the native ECM. Hydrogels are typically classified as natural, synthetic and hybrids. Natural hydrogels include agarose, collagen, fibrin, etc., [45]. Synthetic hydrogels include polyethylene glycol (PEG) and its derivatives such as PEG- diacrylate (PEG-DA) and polylactic acid (PLA), etc. Collagen is a popular hydrogel for mimicking tissue microenvironments, as it is the most common ECM component of the body [46]. Key characteristics of hydrogels include permeability, porosity, biocompatibility, degradability, and binding sites that allow for precise cell attachment, differentiation, and growth. However, some of the major drawbacks of hydrogels include substandard mechanical properties, poor long-term stability, and batch variability. Hydrogels can also serve as membrane barriers that separate channels but allow for diffusion of nutrients and signalling of molecule exchange, permitting close cell–cell contact, crosstalk, and signalling [47,48]. Moreover, ensuring that the materials used for the microfabrication of GOC models allow for crosstalk between the channels is crucial. Moreover, as mentioned prior, ensuring that parameters such as barrier permeability are measured is pivotal.

#### 3.1.1. Sensor Integration

Sensor incorporation has enhanced sensing and instrumentation strategies in GOC models. A key parameter that requires constant monitoring in these models is oxygen level, permitting extended co-cultures under desired conditions. In the recently developed GOC model, perfusable channels have oxygen sensors integrated permitting continuous fine-tuning of oxygen levels at both the basolateral and apical sides of epithelial channels. In the HuMiX model, optodes of 5 mm diameter, with a sensitivity up to 0.03% of O_2_, were bonded into 1.2 mm deep machined pockets using a silicone adhesive, and then cured overnight. Optodes were fixed to both PC enclosures, which were 20 mm adjacent to inlets and outlets of perfusable channels. By this incorporation, authors were able to measure oxygen concentration every 15 min using the sensors, which gave an output to an OXY-4 trace oxygen transmitter/recorder [34].

Similarly, Jalili-Firoozinezhad et al., utilised sensor spots to measure the oxygen concentration in their model. For integration of the sensor spots into their Organ Chip, the authors used a 1 mm biopsy punch to create sensor spots. Then, dipped the discs in uncured PDMS and embedded the discs into the PDMS channel. This was carried out by placing them in moulds at different regions (inlet, middle and outlet), for perfusbale channels, in this case, top and bottom channels. Finally, the authors cured the Organ Chip with the integrated sensors at 60 °C for ~30 min. The two-step moulding process allowed for the accurate placement of the sensors into the relevant regions of interest. Moreover, for the set-up authors used a CCD camera and the VisiSens software that displays the oxygen levels detected by the sensor spots in pseudo colours on a computer. The software calculates oxygen levels through calibration reading defined oxygen levels at 0 and 100% air saturation. For all experiments, the team quantified their oxygen concentration after comparing reading with calibration values [36]. Unfortunately, the number of GOC models with sensors to monitor different parameters are limited and require the use of external techniques for analysis, preventing and delaying real-time decision-making.

#### 3.1.2. Barrier Integrity

 *(a)*  *Transepithelial electrical resistance (TEER*)

**Figure 5:**
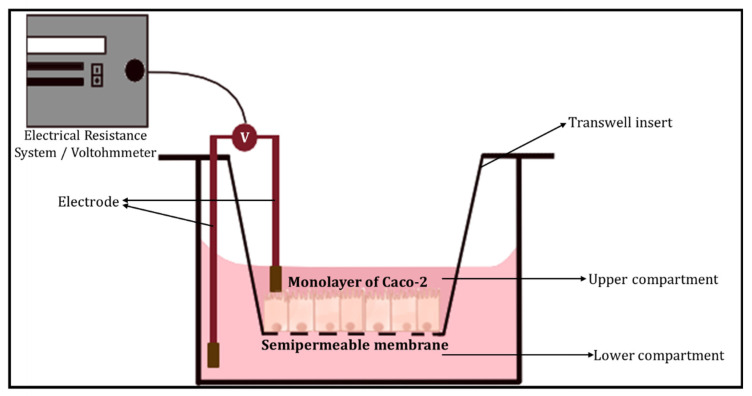
The classic setup used to measure TEER, using an electrical resistance system/Voltohmmeter.

The transepithelial electrical resistance (TEER) measurement is a common quantitative method used to characterise the barrier function of the layers of the cells inside a GOC or OoC models. TEER represents the resistance of electrical current passing through a cellular monolayer, as a measure of the permeability of ions and barrier function. Whereby, the electrical resistance of a monolayer is measured in Ohms. The classic setup is used to measure TEER (Figure 5), where the cellular monolayer is cultured on a semipermeable filter insert and is defined as two compartments: apical or luminal (upper) and basolateral or abluminal (lower). Two electrodes are used and separated by the cellular monolayer, one is placed in the upper compartment and the other in the lower compartment [49,50]. Transepithelial voltage (Vte) and short circuit current (Isc) also provide information about the cellular barrier. All three parameters (TEER, Vte and Isc) are associated with Ohm’s law, as shown below,

(1) $$R=\frac{\;\Delta Vte}{\Delta I}$$

where R represents the resistance (measured in Ohms, Ω), ΔVte is the change in Vte (measured in volts, V) and ΔI is the difference in current flowed (measured in amperes, A). TEER evaluates the electrical resistance across the cell layer and is considered an indicator of the layer’s permeability and robustness [49]. Another method initially determines the resistance across the baseline (R baseline), where there is blank resistance in the membrane (i.e., an absence of cells), then subtracts the readout (R1) which is the resistance across the cell layer on the membrane and multiplies with the growth/surface area of cell culture in cm^2^ (A) as shown below. The final TEER values are expressed as Ω × cm^2^ [51,52].

(2) $$(R_{1}-R_{\mathrm{baseline}})\times A=\mathrm{TEER}$$

Factors other than the robustness of the cell layer are accounted for in TEER measurements, including arrangements of electrodes and resistance in media [32]. For the GI tract TEER values are defined as ‘tight’, ‘intermediate’ or ‘leaky’ represented by values of ~2000, 300–400 or 50–100 Ω cm^2^, respectively.

 *(b)*  *Dextran permeability*

Furthermore, epithelial tight junctions (TJ) create a frontier between the apical and basolateral surface domains of cells, resulting in the regulation of diffusion along the paracellular pathway. The paracellular gate is semi-permeable and restricts diffusion in a size- and charge-selective manner. Barrier integrity can be measured *in vitro* by monitoring molecules of high molecular weight diffused along the paracellular pathway. Specifically, paracellular permeability of hydrophilic ‘tracers’, such as dextran and polystyrene microspheres, that are fluorescently labelled are often monitored. Dextran, a glucose polymer, is the most used due to its suitability for fluorescent labelling as it has an easily accessible carbonyl and hydroxyl group. Dextran can be designed to be of a range of sizes (3–70 kDa) and charges; it is relatively inexpensive, membrane impermeable and non-toxic to cells [53]. Cellular permeability is characterised by better accuracy when utilising different sizes of oligomers. Currently, the predominantly used macromolecules are labelled with fluorophores such as fluorescein isothiocyanate (FITC-dextran), and dextran labelled with rhodamine, etc., [49,54] Moreover, dysfunction of the epithelial barrier results in increased permeability to FITC-dextran and a decrease in TEER [55]. Some limitations of using dextran permeability methods include poor *in vitro* assay robustness, and solutes labelled with non-radioactive fluorophore compounds will not provide sensitivity to show the minute changes in the permeability of the monolayer, due to poor specific activity or fluorophore instability [53].

 *(c)*  *Cellular Junctional Complex Imaging*

Alternately, imaging junctional complex proteins for channels lined with epithelial monolayers in GOC models is an excellent way to ensure barrier integrity. Epithelial cells are bound together by various junctional complexes that are responsible for permeability and barrier integrity. These junctional complexes include TJ, adherens junctions, gap junctions and desmosomes. Whereby, tight junctions are the primary regulators of paracellular permeability, and a complex of proteins situated at the apex of epithelial cells. TJs comprise of numerous transmembrane and cytosolic proteins, such as claudins, occludins, zonula occludens (ZOs), cingulin, tricellulin and junctional adhesion molecules (JAM) [56,57]. Transmembrane proteins such as claudins, occludins and JAM are responsible for linking and sealing the paracellular spaces between adjacent epithelial cells. Claudins, also known as the ‘backbone’ of tight junctions, are differentiated into two classes known as sealing (i.e., responsible for rigidity) and pore-forming claudins. Occludins are commonly associated with controlling intermembrane and paracellular diffusions. Moreover, occludin is a phosphorylated protein, whereby the phosphorylation is associated with TJ localisation [58]. Zonula occluden proteins (e.g., ZO-1, ZO-2 and ZO-3), also known as linker proteins, that connect with other transmembrane proteins such as occludins, claudins and JAM to create strong bonds and interact with the membrane cytoskeleton which consists of F-actin and myosin. ZO proteins essentially form the central system for protein interactions. Tight junction dysregulation results in alterations in barrier function and integrity, often leading to increased production of inflammatory cytokines. Therefore, imaging of junctional complex proteins (zonula occludens-1 (ZO-1) and occludin) is appropriate to measure and determine intact barrier function and integrity [58].

Similarly, the adhesion of endothelial cells is determined by stable interactions between transmembrane proteins that are present in nearby cells. Endothelial cells have namely three types of junctions including gap (i.e., endothelial cells lack desmosomes), tight and adherens junctions. Whereby, vascular endothelial (VE)-cadherin is considered one of the main structures and adhesion protein molecules in adherens junctions, assisting in cell-to-cell contact in endothelial cells. VE-cadherin is responsible for connecting adjacent endothelial cells to each other in a calcium-dependent form. Moreover, VE-cadherin provides the basic organisation of adheren junctions, which is connected through its cytoplasmic domain to β-catenin, p120-catenin and plakoglobin [59]. VE-cadherin can bind intra- and extracellular proteins (as mentioned above) binding molecules assembling into different complexes, that can induce structural changes to the junction and/or initiate intracellular signalling [60,61]. Furthermore, VE-cadherin immunostaining has been performed to investigate the distribution of the adherens junction of a dysfunctional endothelium.

### 3.2. Cell Types

A variety of cell types have been utilised for GOC models. Caco-2 [34] or HT-29 cells have been used to mimic IECs. Caco-2 cells have been used to study intestinal absorption and permeability characteristics for decades. These cells are robust, easily accessible and can spontaneously form crypt and villi structures. Caco-2 cells, due to their resemblance to human intestinal barrier in morphology and polarity, are routinely utilised to predict drug permeability in the intestine [52].

HT-29 cells, commonly used to study the physiology and pathology of human colon cancers, have recently attracted attention because of its ability to express features of mature intestinal cells (e.g., enterocytes). However, compared to Caco-2 cells, HT-29 cells take longer to differentiate (15–20 days in Caco-2 cells versus 30 days in HT-29 cells). The enzymatic activity in HT-29 is lower than that of Caco-2. One major difference between both cell lines, is that HT-29 cells can produce mucin at a relatively higher level than Caco-2 [62]. Tan et al., fabricated a GOC model to study drug transport across the intestinal barrier, utilising Caco-2 cells [38]. The team used immunofluorescence staining to target protein Mucin-2, which positively stained the apical surface of the villous Caco-2 monolayer. The Caco-2 cells reflected no mucus production in static Transwell culture, but Mucin-2 was present under low fluidic shear (~0.008 dyne/cm^2^). The authors suggested that the mucus production was the GI tracts’ defence mechanism against mechanical stress. Shim et al., reported the link between mucus production to high fluid shear flow and peristaltic motion [26]. The team also demonstrated that exposing Caco-2 cells to fluidic shear stress improves human gut’s function by inducing the expression of metabolic enzymes, mucus proteins and the formation of villi-like structures.

Human umbilical vascular endothelial cells (HUVECs) and human intestinal microvascular endothelial cells (HIMECs) are often used to mimic the vasculature [63,64]. Other studies have also used peripheral blood mononuclear cells (PBMCs) isolated from blood as they contain T cells to mimic the immune functions of the vasculature [65,66].

Host immune factors such as antibodies, cytokines, regulatory T cells, etc., are useful indicators of inflammation and thereby inflammatory processes. If the application of GOC model is disease modelling for investigating inflammatory diseases such as inflammatory bowel disease (IBD), immune mediators such as cytokines, their production and stimulation are key parameters to be measured. It assists in understanding the impact of these cytokines on tissue homeostasis. Cytokines are essential in disease pathogenesis. They are small proteins, peptide molecules or glycoproteins that are signalling molecules for complex intercellular interactions. Cytokines are divided into different functional families based on the enhancement of immunological response, including interleukin family (IL), interferon family (IFN), tumour necrosis family (TNF) superfamily and the chemokine family. Cytokines bind to their respective cell surface receptor and initiate a signalling cascade which ultimately results in the up/down regulation of gene expression or transcription factors. The outcome of binding depends on the expression of the complementary receptor, the extracellular levels of cytokine and the type of signalling cascade that is initiated upon binding. Cytokines can either be pro or anti-inflammatory or both, as highlighted in Table 2 [67,68].

Another commonly monitored component in GOC models where the application is disease modelling for specific inflammatory diseases is lipopolysaccharides (LPS). LPS is an endotoxin which forms the outer wall of Gram-negative bacteria. Increased levels of LPS are often noted in inflammatory diseases such as adipose tissue inflammation. In normal physiological conditions, the gut barrier comprising of the mucosal layers and intestinal epithelium minimises the progression of LPS from the bowel into the systemic circulation. Disruption of the gut barrier, due to diet or pathogenic bacteria, leads to LPS displacement and leakiness. Upon entry into circulation, LPS can trigger various signalling pathways and recruit inflammatory cells to the site, including large proteins such as LPS-binding protein (LBP), Toll-like receptor-4 (TLR4), etc. In the lamina propria, the binding of LPS to TRL4 leads to the activation and secretion of proinflammatory mediators resulting in localised inflammation. If LPS enters the bloodstream, it is bound to either LBP or lipoproteins which interacts with surface receptors such as TLR4 found on immune cells. TLR4 itself is not able to bind LPS but requires a cofactor (i.e., CD14), enabling the transfer of LPS to TLR4 and myeloid differentiation factor 2 (MD2), which controls LPS detection. The LBP then transports LPS to CD14, leading to the activation of NF-ĸB, resulting in increased production of proinflammatory cytokines such as TNF-α, IL-6 and IL-1β. Subsequently, macrophages will infiltrate the region, followed by other immune cells resulting in tissue inflammation [70,73].

A common method used to profile the nature of inflammatory responses of GOC models is using transcriptional readouts such as qPCR and RNA sequencing. RNA sequencing is a comprehensive method that provides detailed information about the type of immune response that is induced in the GOC model. Polymerase chain reaction (PCR) can detect mRNA. Flow cytometric assays can identify intracellular proteins. Due to the range and varying location of cytokine activity, the following parameters must be considered: (a) high sensitivity, specificity, and affinity, (b) reproducibility, (c) detection of a variety of cytokines using a small sample, (d) time and cost-effectiveness. Methods for quantifying cytokine production include bioassays, protein microarrays, high-performance liquid chromatography (HPLC), enzyme-linked immunosorbent assay (ELISA), Meso Scale Discovery electrochemiluminescence (MSD) and multiplex immunoassays (MIA). Among these methods, the direct cytokines measurements are centred on immunometric methods such as ELISA and MIA. Briefly, the immunometric method utilises a primary specific antibody, also known as the capture antibody, which is in a fixed position. The capture antibody will bind to the cytokine that is to be analysed. A second antibody (detection antibody) then binds to the cytokine. The detection antibody usually carries a signal-emitting entity (i.e., fluorescence). Upon specific binding, the resulting signal can directly be used to measure the concentration of the cytokine of interest [74,75]. Beaurivage et al., exposed enterocyte-like cells to an immune-relevant inflammatory trigger utilising an OrganoPlate to epitomise critical physiological aspects of IBD, such as the depletion of barrier integrity and increased cytokine production. The authors mimicked the inflammatory state by applying an immune-relevant cytokine trigger that represents the effect of *E. coli*-activated dendritic cells on IECs. The authors assessed the effect of the trigger described above as two main aspects of IBD: the integrity of the intestinal barrier and the cellular activation of IEC. Whereby, the intestinal barrier integrity can be characterised through TEER and the localisation of cell junction-associated E-CADHERIN. The protein expression of IL-1β, IFN-γ and mRNA expression of IFN-γ are upregulated in the mucosa of IBD patients. The team optimised the composition of the trigger, concluding that a combination of IL-1β, TNF-α and IFN-γ resulted in the greatest cytokine production in the Caco-2 cells. The trigger effect was then compared to various concentrations of *E. coli*-activated dendritic cells on the cytokine production of Caco-2 cells. Increased Caco-2 cell activation and a decreased barrier function confirmed the inflammatory state triggered by the immune-relevant cytokines. The cellular activation of Caco-2 cells was quantified by measuring the production of epithelial cytokines (i.e., IP-10, IL-8, and CCL-20). The results indicated an increase in production of the epithelial cytokines upon inflammatory trigger [63].

### 3.3. Stimuli

The human gut experiences both peristalsis and segmentation fluid flow. Both types of stimuli assist in the movement and absorption of foods [76]. Furthermore, in the human intestine shear stress contributes greatly to cell differentiation and allows for greater drug absorption, increased mucus production and elevated mitochondrial activity. Therefore, *in vitro* mechanical stimuli such as shear stress and peristalsis are vital for accurately mimicking the human gut’s physiology, allowing cell differentiation, and preventing bacterial overgrowth [77]. Most GOC models incorporate fluid flow, usually through perfusion channels generating shear stress imitating the human gut. Shear stress in most models is introduced using peristaltic pumps, but these setups are bulky and have low throughput. A key factor imperative for mirroring the peristaltic-like motion is the ability of the cell culture to withstand mechanical deformations over prolonged durations of time. The common range of shear stress within the human gut is between 0.002—0.08 dyne/cm^2^ [15,78]. External pumping and loading systems can accurately reproduce fluid flow rates and cyclical mechanical deformations (i.e., peristalsis) in GOC models with great precision but are bulky and have low parallelisation. Tan et al., overcame the limitation of low throughput with two peristaltic micropumps, each pump had eight pump lines, allowing fluid delivery through the 16 microchannels [38].

Moreover, under continuous flow and cyclic strain Caco-2 cells undergo cell differentiation, polarisation, villi formation, maintenance of barrier integrity, mucus production, etc., compared to static conditions. Specifically, it was noted that Caco-2 cells require less time to differentiate and polarise under continuous flow compared to static conditions. Tan et al., confirmed this by measuring the aminopeptidase activity (i.e., Caco-2 cells which have differentiated express brush border enzymes); at day 5, Caco-2 cells in the microfluidic device expressed notably higher aminopeptidase activity compared to the static Transwell system at day 21. Implying it took less than half time (~16-days difference) for Caco-2 cells to differentiate and polarise under continuous flow compared to static conditions [38]. It was also noted that with the application of cyclic strain Caco-2 cell growth, differentiation and polarisation are accelerated further [79].

Similarly, under static or even very low rates (e.g., 0.5 μL/min) Caco-2 cells are unable to form villi-like structures; however, with the introduction of continuous flow, villi-formation occurs. Tan et al., confirmed this as the Caco-2 cells seeded their device only took ~5 days under continuous flow to form distinct villi-structures [38]. Likewise, Kasendra et al., and Kim et al., also noted similar results suggesting villi-like structures occurred only in the presence of flow [65,79]. Interestingly, others noted villi-formation can still occur in the absence of cyclic strain as observed previously [35,38]. Moreover, Jalili-Firoozinezhad et al., stated with the continuous flow but the absence of cyclic strain, the number of colonised bacteria was remarkably higher (*p* < 0.001) and doubled bacterial cell densities in less than 24 h. They concluded that bacterial overgrowth is significantly increased with the cessation of cyclic strain, emphasizing the importance of incorporating of both fluid flow and cyclic strain [36,65]. Furthermore, other features of Caco-2 cells such as mucus production, well-defined tight junction and barrier integrity were only noted under dynamic flow conditions and were completely absent or depleted in static Transwell cultures [38,65,79].

Furthermore, GOC models should experience both fluid flow and peristaltic-like motion allowing for the GOC model to accurately represent the human gut *in vitro* (Table 3). These dynamic mechanical stimuli not only impact the epithelial cells but also the surrounding microbiota. Moreover, *in vitro* mechanical stimuli such as fluid flow and cyclic strain contribute to epithelial cell differentiation, polarisation, maintenance of barrier integrity, villi formation, mucus production, etc.

### 3.4. Gut Microbiota

The human gut is host to a large variety of microbiomes, which are responsible for maintaining gut homeostasis, nutrient absorption, and drug metabolism. Alterations in the gut microbiome composition disrupt homeostasis, induce pathogenesis and disruptions to the mucosal barrier, leading to bacterial translocation and increasing exposure to pathogens and endotoxins [80].

Jalili-Firoozinezhad et al., used Bacteroides fragilis, human commensal symbiotic bacteria that only grows under aerobic (>0.5% oxygen) conditions. The team isolated the gut microbiota from human faeces [36]. Kim et al., utilised the strain of *Lactobacillus rhamnosus* GG (LGG), originally isolated from the human gut, to study the human intestinal cell-microbe interaction. LGG cells were grown and then transferred to the apical surface of the Caco-2 cells monolayers. To evaluate the viability and function of LGG cells, the team determined the catalytic activity of the LGG β-galactosidases. To do so, the team measured the microbe’s ability to cleave the enzyme substrate, O-nitrophenyl β-d-galactopyranoside. The SpectraMax M5 instrument was used to analyse the collected samples and quantify the amount of product (i.e., O-nitrophenyl) released by the β-galactosidases in the LGG cells. A calibration curve of O-nitrophenyl estimated the amount cleaved [32].

The most established method of characterising gut microbiome was stool sampling. Stool sampling is a non-invasive technique and samples are densely occupied by microbes. Essentially faecal samples were collected, frozen and stored instantly at -80 °C. Subsequently, DNA is extracted from the samples through two stages. First, samples are purified using centrifugation and multiple reagents. Next, samples are incubated with lysis buffer with agitation, allowing further centrifugation. After DNA extraction, the DNA is amplified and 16S rRNA primers are selected for gene sequencing. The resulting gene sequencing data undergo filtering to assure the quality thresholds. Next, operational taxonomic units (OTU) analysis is undertaken. Before this, sequence counts are normalised. The OTU is a method through which related bacteria are categorised and grouped [70].

Monitoring the oxygen supply and concentration is crucial to maintain the gut-microbiome ecosystem. Luminal oxygen levels below 0.5% are necessary since most commensal bacteria are anaerobic. Electrochemical, optical and laser methods can measure the oxygen concentration. Electrochemical and optical are commonly used for measuring dissolved oxygen (DO) levels in liquid. For low intestinal oxygen levels, optical sensors have been advantageous as they prevent an oxygen depletion region from forming and do not require direct contact with the solution [15]. Optical sensors are light-based sensors that measure the change in wavelength after the interaction of an analyte with the bio-recognition element [81]. The most common optical sensors are fluorescent and plasmonic sensors [82]. HuMiX was one of the first GOC models incorporating oxygen sensors. The model has four pst3 oxygen sensors (optodes) affixed to deep-machined pockets of the PC enclosure using silicone adhesive. The simultaneous perfusion of anoxic media (0.1%) through the microbial microchamber, allowed the oxygen level to be maintained at 0.8%. Moreover, the integration of the optodes allowed for the continuous detection and real-time monitoring of DO concentration in the HuMiX model [34].

Another GOC model incorporating high-resolution dissolved oxygen monitoring is the Intestine Chip, which had six sensor disks with oxygen-quenched fluorescent particles fixed on the top and bottom sections of the model, allowing for real-time monitoring of oxygen levels. This GOC model has a central anaerobic chamber which is frequently flushed with saturated 5% CO_2_ nitrogen gas, sustaining low oxygen levels within the lumen in the upper chamber. Oxygen concentration is determined by variations in fluorescence intensity produced by the sensors, which are captured with the VisiSens camera [36].

## 4. Applications of GOC Models

**Figure 6:**
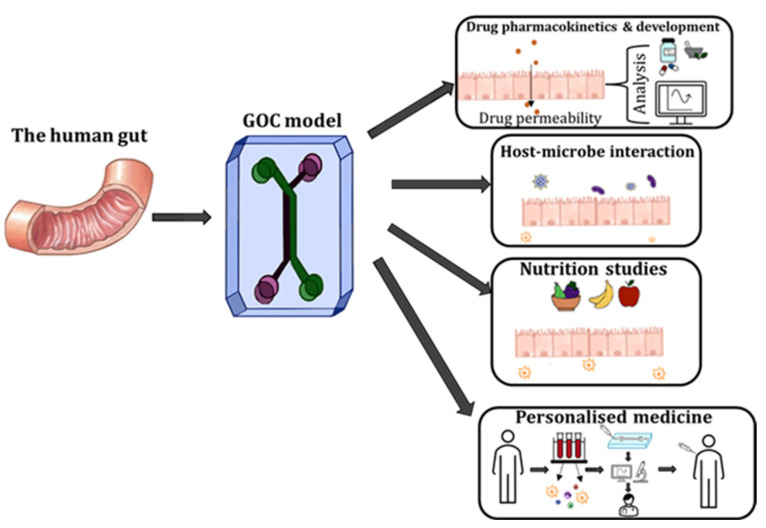
The various applications that GOC models can be utilised, including drug pharmacokinetics and development, studying host-microbe interactions, nutrition and metabolism studies and personalised medicine.

GOC devices have been rigorously applied to study cellular- and tissue-level interactions and relationships *in vitro*. Moreover, GOC is a powerful tool for studying the physiology of the gut, conducting drug testing and development, as well as investigating host-microbiome interactions. Other relevant applications of GOC models include understanding the correlation between host-microbe, as well as human nutrition and human microbiome (Figure 6) [12,83].

There has also been growing interest in utilising OoC models to study tissue–tissue interfaces. For example, gut-brain-axis (GBA) chips have been developed and employed to further understand how alterations in gut physiology or microenvironment caused due to diet, medication, etc., may result in physiological changes in the brain or neurodegenerative disorders [84,85,86]. Likewise, gut-liver-chips have been developed to further study the intestinal barrier under variant conditions. Such as to understand the correlation between hepatic dysfunction and barrier permeability, the development of liver disorders by alteration in gut microbiota composition, etc., [87,88,89]. Another OoC model, a chip that collated two systems, the GI tract, and the liver, to further mimic and understand human metabolism accurately. Interestingly this system is pumpless, utilising gravity to drive fluidic flow. Moreover, the authors claim this chip can be utilised for drug testing and development [90,91]. Trapecar et al., developed a gut-liver-brain chip, essentially a physiomimetic model, which was used to mimic the early onset of Parkinson’s disease (PD). Moreover, this team was able to incorporate both innate and adaptive immune cells into their systems. Authors also used hiPSCs from a donor with familial PD, allowing them to partly recreate a clinical manifestation of familial PD [92].

A common application of GOC models is the detailed investigation of host-microbe interactions. For instance, Kim et al., developed a GOC model that investigates the effect of peristalsis and shear stress on the epithelial cell layer in the intestine. The team aimed to create a system closely resembling the human intestine by incorporating gut microbes and dynamic flow as experienced in the human intestine. This system consists of two channels, separated by a porous membrane that was coated ECM. The team utilised Caco-2 cells in their model to represent the intestinal epithelial cells (IECs). These cells experienced peristaltic motion, assisting in phenotypic and expression modifications. The team also evaluated the ability to maintain intestinal microbes. *Lactobacillus rhamnosus GG* (LGG), commonly used to study human intestinal cell–microbe interaction, was co-cultured on the luminal surface of the cultured epithelium for a week, without compromising epithelial cell viability. Authors were able to maintain a co-culture of intestinal cell monolayer with normal barrier function and microbes growing on its apical surface, and barrier integrity was also sustained and later improved. Authors noted that LGG that was tightly adherent to the surface of Caco-2 cells remained in the model under continuous flow compared to non-adherent LGG, which was washed out preventing overgrowth. Furthermore, authors also established that in the presence of LGG, co-culture intestinal epithelial integrity was increased. This was the case because the microbe provides a normal microenvironmental cues which in turn increases the cellular function of the epithelial cells (i.e., mucin secretion). This GOC model epitomised various dynamic flow, phenotypic and function features of the human intestine. For the first time, the model could integrate both shear flow and peristaltic motion on the intestinal epithelial layer. Both features have hence forth been deemed crucial for the accurate recapitulation of the human intestine. Authors believe this GOC model will facilitate the study of host-microbes symbiotic relationship [32,93].

Furthermore, Kim et al., reported another GOC model for studying the interactions and correlation between human intestine, immune system, and bacteria. The study mainly aimed to develop a GOC model for human intestinal inflammation and bacterial overgrowth. The results ultimately allow for the *in vitro* analysis of contributors to the pathophysiology of inflammatory intestinal diseases such as IBD, a period. The authors aimed to analyse how bacteria (i.e., probiotic, and pathogenic), lipopolysaccharides (LPS), immune cells, vascular endothelial cells, inflammatory cytokines and mechanical stimuli contribute to intestinal inflammation and thereby jeopardise the integrity of the epithelial barrier. The team explored whether they could replicate the protective effects of clinical probiotics and antibiotic therapies in the GOC model, utilising the model as a potential tool for drug development and studying the pathophysiology and disease mechanisms. This system reproduced dynamic flow, and phenotypic and functional features of human intestine and recreated an inflammatory response that accurately represents *in vivo* conditions. The results demonstrated that the integration of immune cells (i.e., peripheral blood mononuclear cells (PBMCs), lead to villus injury and a decline in intestinal barrier integrity. Moreover, similar result was obtained in the presence of bacteria (commensal and/or pathogenic) and PBMCs. The authors suggested that intestinal barrier integrity and villi structure were both compromised by the incorporation of bacterial endotoxins (i.e., LPS and immune cells) [65].

HuMiX, a more recently developed GOC model, allows for the co-culture of human and microbial cells under *in vivo* conditions. The HuMiX model consists of three stacked elastomeric gaskets sandwiched between two polycarbonate (PC) enclosures. The top channel is the microbial microchamber, which contains the bacterial biofilm. The middle channel hosts a monolayer of Caco-2 cells. The bottom channel is the perfusion chamber, which perfuses culture media into the middle channel. The team was able to recapitulate *in vivo* transcriptional, metabolic, and immunological responses in human IECs with co-culture with commensal LGG under anaerobic conditions. The HuMiX models’ can co-culture human and microbial cells and allow for systematic investigations of host–microbiome interactions. Furthermore, HuMiX can also be used in the future for drug discovery, screening, delivery, and nutritional studies [34].

## 5. Limitations and Prospects of GOC Models

Microfluidic GOC and OoC models have become a popular research topic, as they faithfully mimic the various characteristics and responses as seen in physiology. Unfortunately, there are still significant limitations that affect the accuracy of these models. For example, GOC models have omitted features that contribute significantly to the physiology and pathology of disease models.

A commonly noted limitation in GOC models is the absence of supporting cells and tissues typically found in the human intestine. For instance, the inclusion of the microvascular endothelium, immune cells, muscle cells or enteric nerves is pivotal for accurately representing the human gut and its complex functions [31]. For example, smooth muscle cells surrounding the GI tract are crucial for peristaltic motion (i.e., propulsion of food through the intestine), allowing for the efficient digestion of food and absorption of nutrients. The enteric nerves are vital for functions such as movement throughout the GI tract, regulation of gastric acid secretions, regulating blood flow and maintaining the gut barrier [94]. Absence of these supporting cells results in the GOC models not accurately comprising the real complexity of the human gut and its functions missing.

Some fabrication drawbacks of GOC models include alternative material instead of PDMS and sensor integration. The short lifespan of cell lines has been often associated with the biomaterials used in GOC models. An important consideration for GOC models used commercially or clinically, e.g., drug testing, microfluidic systems must use biomaterials that are biocompatible, non-absorbent, and inert. The biomaterials used for the system should also avoid introducing by-products of other materials into the system, causing possible contamination. As mentioned earlier, PDMS is the commonly used material for microfluidic OoC models; however, PDMS has drawbacks including its difficulty for cell adhesion and ability to absorb small or hydrophobic molecules. To overcome this limitation, PDMS surface modifications can be done by coating with ECM proteins or hydrogel to prolong cell adhesion and survival. Furthermore, the use of stimuli-responsive biomaterials has gained attention, although none have been implemented in GOC models [95,96]. Materials such as glass and polymethyl methacrylate (PMMA) are also beneficial but are often not used or avoided due to manufacturing difficulty. Interestingly, Off-Stoichiometry Thiol–Ene (OSTE) has gained attention as a novel polymer platform that can be used for the fabrication of microfluidic devices. Future work with GOC models should investigate the possible replacement of PDMS with OSTE. As mentioned prior, the incorporation of sensors in GOC models is often overlooked, but it is vital for accurate and real-time analysis permitting real-time decision-making. For example, instead of TEER measurements, the use of mass spectrometric analysis could be used to assist in the identification and quantification of biological and chemical compounds and stimuli [97,98]. Moreover, there has been growing interest in label-free optical based read-out techniques, such as surface-enhanced Raman spectroscopy (SERS). SERs, due to its biocompatibility, high sensitivity and selectivity is viewed as a powerful tool that can be utilised for biological analysis and assess complex reactions. Furthermore, SERS is a promising tool that can be adapted for GOC models [99,100,101,102].

In addition, another drawback of existing GOC models is the limited lifespan of the cells and cell sources in the devices. This challenge is exacerbated when using primary cell lines compared to immortalised cell lines. Unfortunately, immortalised cell lines are also limited in faithfully mimicking *in vivo* conditions. Caco-2 cells are cultured for usually 21 days to achieve a polarised monolayer that fully expresses tight junctions and other intracellular contacts. However, when they are utilised for GOC models, Caco-2 cells cannot grow for extended periods due to the presence or incorporation of microbes [52]. Incorporating induced pluripotent stem cells (iPSC)-derived cells from humans has been considered for GOC and OoC models to assist in generating a more relevant differentiated human cell. iPSC recently has gained attention for drug development and disease modelling. However, there are limitations such as ethical challenges surrounding the use of iPSCs, lack of robust differentiation protocols and inconsistent efficiency [12]. Another possible contributor to short lifespan of cell lines could be biomaterials used in GOC models.

Another limitation is the absence of a mucus layer. The GI tract is covered with a protective mucus layer composed mostly of mucins secreted by goblet cells. The mucus layer is essential for maintaining homeostasis, protection against pathogens and selective transport [103]. Therefore, the inclusion of a mucus layer accurately models the human gut and creates a simulation of chemical and physical interactions between commensal microbes and IECs. When the mucus layer is present, IECs produce high concentrations of antimicrobial molecules that prevent any harmful effects of bacterial growth and colonisation. Mucin production is tissue and cell-type specific and affected by differentiation, and mucus is composed of ~50 mucins. Mucin-2 (MUC2) is produced predominantly by goblet cells and is considered the major constituent of the mucus layer, protecting the IECs from pathogens and toxins. Caco-2 cells, which are the most utilised cell line for GOC models, under static conditions do not secrete mucus, but can produce mucus under flow and cyclic strain emulating peristaltic motion. Polarised Caco-2 cells do not express MUC2, rather mainly secrete transmembrane mucins such as mucin 3 (MUC3), mucin 12 (MUC12) and mucin 17 (MUC17) and cannot secrete thick mucin layers unless stimulated by growth factors that stimulate goblet cell differentiation [104,105]. Under dynamic culture conditions, both fluid shear stress and cyclic strain that mimic peristaltic motion, result in Caco-2 differentiation leading to four main cell lineages (i.e., absorptive (enterocytes), mucus-secretion (goblet cells), enteroendocrine (EECs) and Paneth) [106,107]. Applying this, previous GOC models [14,32,38] utilised Caco-2 cells to produce MUC2 by allowing Caco-2 cells to differentiate into goblet cells upon stimulation by flow and cyclic strain. However, this may not always be successfully achieved. An alternative is co-culturing HT29-MTX, and Caco-2 cell lines. Co-culturing these cell lines can not only accurately mimic the permeability features and cell diversity of the GI tract, but also will assist in mucin production. The ratios of Caco-2 and HT29-MTX used to represent the small and large intestine are ~90/10 and ~70/30 or 75/25, respectively [108,109].

Another component often overlooked is the absence of microvilli, which is essential to model the architecture of the gut. Previously, numerous studies have used micro moulding and unique engineering approaches to form polymeric scaffolds (e.g., collagen gel) into villus-shaped structures [31,110]. Some drawbacks have been noted with this model due to it blocking the abluminal surface of the epithelium, thereby halting absorption [15]. Alternatively, Shim et al., developed collagen-based villi-like structures via soft lithography [26]. A similar method was adopted by Costello et al., where polyethylene- vinyl-acetate (PEVA) was used to 3D print a scaffold that resembled intestinal topography, which was then incorporated into a bioreactor, where cells were exposed to flow. This approach allowed for a longer culture of up to 32 days [111].

Furthermore, the incorporation of gut microbiota to accurately represent human physiology has not been achieved successfully. The human gut harbours a large complex microbiome, ~100 trillion, between 500 and 1000 species. The present GOC models are not able to incorporate all the prominent families of intestinal flora. Moreover, it has been proven challenging to co-culture complex microbial species and IECs in the GOC model due to diverse media and oxygen conditions [112]. Therefore, an important consideration for future GOC models is to develop a robust protocol that facilitates the co-culture of aerobic host cells and anaerobic microbes for extended time periods.

Another challenge in the application of GOC is modelling disease-specific phenotypic characteristics and angiogenesis. Beaurivage et al., integrated patient-derived human primary material, IECs with monocyte-derived macrophages to model the human intestine and create a disease model for IBD. The inflammatory state of the co-culture was induced by LPS derived from *E. coli* and recombinant human interferon-gamma (RH IFN-gamma) and was confirmed by RNA-sequencing of human intestinal organoids (HIO). The authors successfully characterised the inflammatory phenotype as seen in IBD, by pinpointing cell-specific cytokine production. Since IBD is a multi-factorial disease, authors recognised that one cell type cannot represent the disease, therefore macrophages were introduced into the model to increase its complexity. Although this model incorporated immune components relevant to the disease, the model lacked the presence of microbes and microvascular endothelial cells, components that are essential to represent an accurate human intestine in a GOC model [64]. An important consideration when representing disease states *in vitro* is the inclusion of relevant components such as surrounding cell types, presence of immune cells, presence of microbes, etc., as mentioned prior. Researchers can provide precise and personalised diagnostics and medicine by using relevant immune cells and organoid culture derived from primary patient intestinal cells and gut microbiome.

## 6. Conclusions

In the past decade, extensive work and advances were made in the research area of GOC and OoC systems to accurately emulate complex systems such as intestinal physiology. Although these systems have yielded great innovations, which expanded our understanding of the relationship of complex systems (i.e., host-microbe interactions), the body of work also indicated immense difficulty in mimicking human-specific features *in vitro* as technical challenges remain. Nonetheless, in the future, with the optimisation and incorporation of microfluidic designs and key cellular components, GOC technology can be employed for advanced host-microbe research, drug pharmacokinetics, nutrition studies, etc. Most importantly GOC technology can be a powerful tool that can be harnessed for personalised medicine, by utilising patient samples (i.e., immune cells, microbiome, etc.,), testing and monitoring patient-specific response to medication.

## Figures and Tables

**Table 1:** Summary of technological approaches and characteristics of representative GOC models.

GOC Model	Device Material	Configuration	Membrane Properties	Intestinal Cell Type	Microbes Co-Culture	Shear Stress	Cyclic Strain	Oxygen Gradient
HuMiX [34]	Polycarbonate (PC)	3 co-laminar channels (stacked)	Microporous membrane pore diameter = 1 µm Nanoporous membrane pore diameter = 50 nm	Caco-2	*Lactobacillus rhamnosus* GG (LGG)	Yes	No	Yes
Organ-on-Chip with TEER [35]	PC/PDMS	2 layered channels	Pore size = 10 µm	Caco-2	-	Yes	No	No
Intestine Chips [36]	PDMS	2 layered channels	Pore size = 10 µm	Caco-2	*B. fragilis* & for microbiota co-culture, colon and cecum content from five mice colonised with healthy human microbiota (Hmb)	Yes	Yes	Yes
GOC model [32]	PDMS	2 layered channels	Pore size = 10 µm	Caco-2	*Lactobacillus rhamnosus GG*(LGG)	Yes	Yes	No
Peristaltic Human Gut-Vessel Microsystem [37]	PDMS	3 layered channels	Pore size = 10 µm	Caco-2	*Escherichia coli*	Yes	Yes	No
Thiol-ene microchip [38]	PDMS	2 layered channels	PTFE pore size = 0.4 μm	Caco-2	-	Yes	No	No

**Table 2:** Cytokine family divided into functional families based of inflammatory nature of cytokine.

Pro-inflammatory:	IL-1β IL-7 IL-8 IL-12 IL-15 IL-17 IL-18 IL-23 IL-33 IL-34 G-CSF TNF- α TNF- β IFN- γ
Anti-inflammatory:	IL-4 IL-5 IL-10 IL-13 IL-22 IL-27 IL-35 IL-37 (IL-1F7) IL-38 (IL-1F10) TGF-β
Variable:	IL-6 * IL-11 * IFN-α * IFN- β *

Abbreviations: IL; interleukin, IFN; interleukin, G-CSF; granulocyte colony stimulating factor, TNF; tumour necrosis factor, TGF; transforming growth factor. * Have contrasting mechanisms suggesting that the cytokine may be involved in both pro- and anti-inflammatory activities [69,70,71,72].

**Table 3:** Effect of dynamic fluid flow on different GOC models and respective cell lines.

GOC Model	Flow Rate:	Outcome:
HuMiX [34]	Flow rate: 25 µL min ^−1^ (Shear rate not reported)	Caco-2 cell growth and differentiation Allowed for constant monitoring of the effects of the co-culture on the individual co-cultured cell contingents.
Organ-on-Chip with TEER [35]	Shear rate: 1 dyne/cm^2^ (equivalent to 60 µL h^−1^)	Caco-2 cells differentiation Spontaneous villi-formation
Intestine Chips [36]	Flow rate: 60 µL h^−1^ (Shear rate not reported) Cyclic strain: 10% cell strain; 0.15 Hz frequency	Caco-2 cell growth, differentiation, and polarisation Spontaneous villi-formation Mucus production and established barrier function.
Intestine Chip [79]	Flow rate: 60 µL h^−1^ (Shear rate not reported) Cyclic strain: 10% cell strain; 0.2 Hz frequency	Epithelial cell growth and differentiation Well-defined intestinal folds. Confluent monolayers with well-developed tight junctions and barrier functions
GOC model [32]	Shear stress: 0.02 dyne cm^2^ (equivalent to flow rate of 30 μL h^−1^) Cyclic strain: 10% cell strain; 0.15 Hz frequency	Epithelial cell differentiation. Spontaneous villi-formation
Peristaltic Human Gut-Vessel Microsystem [37]	Shear stress: 0.04 dyne/cm^2^ (equivalent to flow rate of 60 μL h^−1^) Cyclic strain: 15% cell strain, 0.15 Hz frequency	Caco-2 cell growth, differentiation, and polarisation Spontaneous villi-formation Confluent monolayers with well-developed tight junctions and barrier functions Increase in the secretion of glycocalyx
Thiol-ene microchip [38]	Shear stress: 0.008 dyne/cm^2^ (equivalent to flow rate of 3 μL/min)	Caco-2 cell growth, differentiation, and polarisation Spontaneous villi-formation Confluent monolayers with well-developed tight junctions and barrier functions

## Data Availability

Not applicable.
